# SARS-CoV-2 Vaccine-Induced Humoral Immunity in Immunocompetent European Adults: A Systematic Review

**DOI:** 10.3390/microorganisms13030535

**Published:** 2025-02-27

**Authors:** Izabella Bylica, Estera Jachowicz-Matczak, Justyna Brodowicz, Joanna Sułkowska, Monika Bociąga-Jasik, Piotr Heczko, Sebastian Gagatek, Jan Bylica, Jadwiga Wójkowska-Mach

**Affiliations:** 1 Department of Microbiology, Faculty of Medicine, Jagiellonian University Medical College, 31-066 Krakow, Poland; estera.jachowicz-matczak@uj.edu.pl (E.J.-M.); piotr.heczko@pebeha.pl (P.H.); jadwiga.wojkowska-mach@uj.edu.pl (J.W.-M.); 2 Doctoral School of Medical and Health Sciences, Jagiellonian University Medical College, 31-066 Krakow, Poland; jan.bylica@doctoral.uj.edu.pl; 3 Department of Clinical Biochemistry, Faculty of Medicine, Jagiellonian University Medical College, Skawinska 8, 31-066 Krakow, Poland; justyna.brodowicz@uj.edu.pl; 4 Students’ Scientific Group of Microbiology, Faculty of Medicine, Jagiellonian University Medical College, 31-066 Krakow, Poland; joasia.sulkowska@doctoral.uj.edu.pl; 5 Department of Infectious Diseases and Tropical Medicine, Department of Infectious Diseases, Jagiellonian University Medical College, 31-066 Krakow, Poland; monika.bociaga-jasik@uj.edu.pl; 6 Department of Medical Sciences, Respiratory, Allergy and Sleep Research, Uppsala University, 751 85 Uppsala, Sweden; sebastian.gagatek@akademiska.se; 7 Department of Internal Medicine and Gerontology, University Hospital in Krakow, Jakubowskiego 2, 30-688 Kraków, Poland

**Keywords:** vaccination, COVID-19, mRNA vaccines, antibody levels

## Abstract

The COVID-19 pandemic, caused by SARS-CoV-2, profoundly impacted global health systems and economies. Vaccination and diagnostic advancements were pivotal in managing the pandemic. This systematic review evaluates antibody levels in adults following complete COVID-19 vaccination and examines the prevalence of infections in vaccinated populations. A systematic review adhering to PRISMA guidelines was conducted, focusing on studies analyzing antibody levels at least 14 days after full vaccination with FDA- or EMA-approved vaccines. Five European studies meeting the inclusion criteria were selected. Data were extracted and synthesized from studies involving 6280 participants aged 19 to 105, with an average of 11% having prior exposure to SARS-CoV-2. Antibody levels were analyzed over time, and the incidence of post-vaccination COVID-19 cases was recorded. The reviewed studies demonstrated that antibody levels peaked shortly after vaccination but gradually declined over time. Individuals with prior SARS-CoV-2 infection exhibited higher antibody titers than those without prior exposure. After the first dose, the Pfizer–BioNTech vaccine led to significantly higher antibody levels than the Oxford–AstraZeneca vaccine, especially in those without prior infection. Across all studies, the incidence of COVID-19 among vaccinated individuals was low (0.1–3.8% for 144–302 days post-vaccination). Vaccination reduced severe outcomes despite decreasing antibody levels. The decline in new COVID-19 cases and related deaths is attributed to widespread vaccination, natural immunity, and virus mutations reducing severity. Further studies are warranted to explore antibody persistence and optimal vaccination strategies.

## 1. Introduction

Recently, the world witnessed the development of the COVID-19 pandemic, caused by the new coronavirus SARS-CoV-2. This pandemic was particularly dangerous as the virus caused a substantial number of infections and deaths worldwide, overwhelming healthcare systems and putting immense pressure on medical resources. Additionally, it had a profound and far-reaching impact on the global economy, primarily due to widespread lockdowns that disrupted industries. Different countries adopted varied approaches to their COVID-19 policies. Some implemented strict lockdowns and extensive testing regimes, while others focused on achieving herd immunity by naturally acquired infection and prioritized keeping their economies open. For example, in March 2020, when many European countries, including Poland, decided to implement lockdowns, Sweden issued recommendations to maintain hygiene, avoid social contact, and limit movement without closing educational institutions [1]. Differences in epidemic regulations have been reflected in epidemiological data. In Poland, in 2020, 3384 cases of COVID-19 per 100,000 inhabitants were recorded, along with 75 deaths per 100,000 inhabitants. Meanwhile, in Sweden, there were 5955 cases of COVID-19 per 100,000 inhabitants and 95 deaths per 100,000 inhabitants [2,3,4]. Thus, before the development of specific vaccines and antiviral drugs, the spread of the COVID-19 pandemic was determined only by differences in socioeconomic levels and healthcare organization in various countries [5].

The COVID-19 epidemiology changed dramatically when new vaccines against the SARS-CoV-2 virus were developed and licensed and the vaccination campaign was introduced [6].

Therefore, there was an urgent need to develop vaccines and antiviral drugs. The development of anti-SARS-CoV-2 vaccines marked a critical and groundbreaking milestone due to their swift creation and approval during the COVID-19 pandemic. This was made especially possible by the mRNA technology, which enables the intracellular production of the target vaccine antigen [7]. After injection, the lipid nanoparticles deliver the mRNA into cells, typically muscle cells at the injection site. Once inside the cell, the mRNA is read by the ribosomes. This process generates the target protein (e.g., the viral spike protein) [7]. The produced protein is then displayed on the cell’s surface or released into the body. The immune system recognizes this protein as foreign and responds by producing antibodies that can neutralize the virus if encountered in the future and activating T cells that help destroy infected cells and coordinate the immune response [7]. Altogether, available vaccines against COVID-19, approved by the EMA and FDA, were, except for mRNA vaccines, classical vector vaccines and inactivated vaccines.

Vaccination according to the basic schedule and subsequent booster doses were recommended for all vaccines [8]. On 31 December 2022 in Europe, a total of 268,677,438 vaccine doses were administered [9]. Immediately after the vaccinations started, levels of the specific antibodies were followed in vaccinated populations, which resulted in high numbers of published studies [10,11].

There are three basic types of tests in the diagnosis of SARS-CoV-2 infections: molecular, antigenic, and serological. Molecular tests detect the infection at the earliest stages, while those based on the detection of antigens and antibodies provide reliable results later, due to the occurrence of the so-called serological window. In the case of COVID-19, this period is 14 days.

Starting from January 2023, the number of new COVID-19 cases and related deaths significantly declined [12]. However, the question arises whether the worldwide process of vaccination contributed to the termination of the pandemic, and whether it was related to a significant increase in specific antibodies’ levels. To approach an answer to this question, this review was conducted. Thus, the objective of this systematic review was to assess the antibody levels in adults at various time points following full vaccination (defined as a minimum of two doses for most vaccines or a single dose for the Jcovden vaccine) and period prevalence in the vaccinated population.

## 2. Materials and Methods

**Figure 1:**
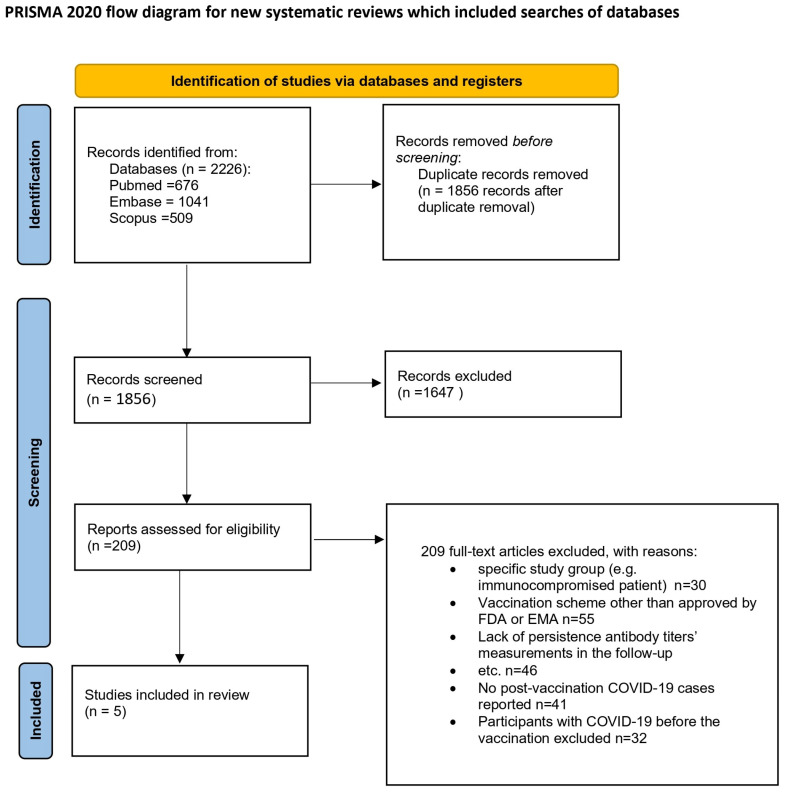
Flow diagram [14]. This work is licensed under CC BY 4.0. To view a copy of this license, visit https://creativecommons.org/licenses/by/4.0/ (25 January 2025).

This review was carried out according to the Preferred Reporting Items for Systematic Reviews and Meta-Analyses (PRISMA). The review protocol was registered in the PROSPERO database with the number CRD42022383367 [13]. Figure 1 depicts the process of preparing for the review.

### 2.1. Searches

The methodology for this systematic review began with a thorough search of electronic databases including Embase and MEDLINE/PubMed, conducted on 4 January 2023. The search strategy was structured to include relevant terms related to COVID-19 or SARS-CoV-2, antibody levels or immunogenicity, and vaccines or vaccination. The full search query can be found in the Supplementary Materials (Table 1).

### 2.2. Types of Included Studies

Notably, this review specifically excluded systematic reviews and meta-analyses. Following the primary electronic searches, a manual screening of citations and reference lists from the included manuscripts was conducted to ensure comprehensive coverage. Only studies published in English and involving human subjects were considered. The inclusion criteria focused on studies analyzing antibody levels (examined by a validated diagnostic test—Supplementary Material Table S1) at least 14 days after complete vaccination using FDA- or EMA-approved vaccines. Exclusion criteria encompassed studies involving participants under 18, those with immunodeficiency, individuals not vaccinated post-COVID-19, secondary analyses, case series/reports, non-English studies, studies lacking quantitative antibody analysis, those focusing on specific comorbidities, and populations such as pregnant women or the elderly over 80 years old.

### 2.3. Data Selection

In this study, data selection was conducted utilizing the systematic review software Cadima [15]. A total of 2226 records were retrieved during the search process. Two pairs of reviewers (I.B. with J.B., and E.J.-M. with J.S.) independently screened the titles and abstracts against predefined inclusion/exclusion criteria, resulting in the identification of 145 potentially relevant studies. Subsequently, the full texts of these studies were examined, leading to the final selection of 5 studies. Any discrepancies during the selection process were resolved by a third reviewer (I.B. or E.J.-M.). Excluded studies (n = 140) were documented along with justifications. This study’s selection process is depicted in a PRISMA flow diagram (Figure 1).

### 2.4. Evaluation of Quality

The methodological quality of the included studies was assessed using Joanna Briggs Institute (JBI) critical appraisal tools tailored to various types of primary studies. This evaluation aimed to gauge the trustworthiness, relevance, and results of the published papers by scrutinizing their methodological rigor and addressing potential biases in design, conduct, and analysis.

### 2.5. Comparison of the Results

In order to compare the number of COVID-19 cases during the follow-up, we calculated the period prevalence rate [%], defined as (the total number of new COVID-19 cases during the observation period/the studied population) and expressed as a percentage. Certain data obtained by the authors were not provided in our summary as they are unrelated to the objectives of the study, such as antibody levels before receiving the full dose of the vaccination. In our article, the units were converted based on the conversion factor [AU/mL] * 0.1428 = [BAU/mL] and [U/mL] = [BAU/mL] [16]. In order to compare all SARS-CoV-2 antibody levels, the IgG, IgM, and IgA classes were converted into BAU/mL in the five evaluated articles (Table 2).

## 3. Results

**Figure 2:**
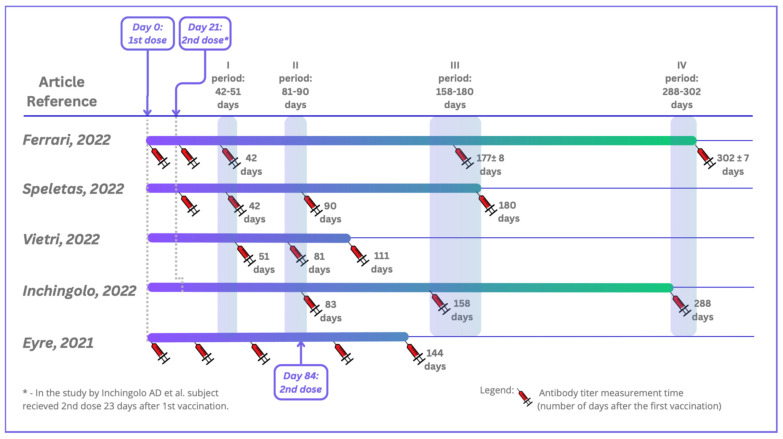
Timeline depicting serum sample collections in the included studies. Ferrari D et al. (2022) [17]; Speletas M et al. (2022) [18]; Vietri MT et al. (2022) [19]; Inchingolo AD et al. (2022) [20]; Eyre DW et al. (2021) [21].

Five studies were included in this systematic review in accordance with both the inclusion and exclusion criteria. All of the studies were conducted in Europe: three in Italy, one in Greece, and one in the United Kingdom. They were carried out between 12.2020 and 10.2021. The total number of patients who participated in the included studies was 6280, and they were of both sexes and aged between 19 and 105. On average, 11% of the participants had previous contact with the virus SARS-CoV-2 (Table 3). The studies were performed using four different serological tests for detecting specific antibodies, including rapid cassette tests based on lateral flow immunochromatographic assays (LFIAs), electrochemiluminescent assays (ECLIAs), chemiluminescent microparticle immunoassays (CMIAs), automated chemiluminescence tests, Clinical Laboratory Improvement Amendments (CLIA), and conventional enzyme-linked immunosorbent assays (ELISAs), using both qualitative and quantitative assays. The tests detected IgG antibodies, and in one study also IgA antibodies. Blood from patients was collected just before vaccination (one study), 21 days after vaccination (five studies), and at subsequent time points (up to 9 months after the second dose of vaccine at the latest). A comparison of the serum sample collections in the selected studies is shown in Figure 2. Over time, patients’ antibody levels decreased slightly in each study, especially for participants without SARS-CoV-2 infection before vaccination (Table 2).

Importantly, the studies included only basic vaccination schedules without booster doses. The most frequently administered preparation was the mRNA Pfizer–BioNTech (BNT162b2) vaccine. Among the five studies, in four, all participants received the BNT162b2 vaccine, and in one study, 66% of the participants were vaccinated with BNT162b2 and 34% with the Oxford–AstraZeneca vaccine. Regardless of the preparation administered and the age of the patients, only a few people had COVID-19 after vaccination: a total of 34 people out of 6,280. The period prevalence rates for the five included studies ranged from 0.1 to 1.3 per 100 person-months; the specific rates were as follows: 0.2 [17,18], 1.3 [19], 0.05 [20], and 0.1 [21].

It should be stressed that this review was based on a very limited number of studies based on humoral antibody responses to a SARS-CoV-2 antigen—the spike protein. Moreover, reports on virus-neutralizing antibodies were not included, due to the lack of papers involving these antibodies and meeting all the inclusion criteria.

In the study by Ferrari et al. [17], serum samples were collected from patients four times. The first collection took place just before vaccination. After testing the samples, the patients were divided into two groups—COVID-19-positive and COVID-19-negative. The second sample collection took place 21 days after the first vaccine dose (at the moment of the second dose). In the COVID-19-positive group, 87.8% of HCPs showed titers above 2500 U/mL, but the median value of the whole COVID-19-negative group was 32.3 U/mL. Another collection 42 days after the first dose showed that in the COVID-19-positive group, 95.9% of HCWs had antibody titers above the 2500 U/mL. The median antibody titer of the COVID-19-negative group increased to 1659.0 U/mL. The third collection took place 177 days (±8 days) after the first vaccination. In the COVID-19-positive group, the median value of the antibody titers was 1288 U/mL, and in the COVID-19-negative group, the median value of the antibody titers dropped to 584.0 U/mL The last collection took place 302 ± 7 days after the first collection. In the COVID-19-positive group, the median value of the antibody titers was 2308 U/mL, and in the COVID-19-negative group, the median value decreased to 419 U/mL. In general, the antibody levels increased after full vaccination and then declined over time. All individuals received the BNT162b2 vaccine.

In Speletas et al.’s [18] study, blood samples were collected four times: day 21 after vaccination directly before the second vaccination, day 42, day 90, and day 180 after the first vaccination. All individuals received the Pfizer–BioNTech (BNT162b2) vaccine. Depending on whether patients had been exposed to the virus or not, the average level of their antibodies during the study varied. However, in both groups, the antibody levels decreased over time after vaccination. The median IgG level was 17,353.0 AU/mL for the COVID-19-infected group vs. 364.8 AU/mL for the non-infected group (21 days after vaccination). Ninety days after the first vaccination, the median IgG levels were 29,646.0 AU/mL (COVID-infected group) and 7593.0 AU/mL (non-infected group). Also, at 90 and 180 days after the first vaccination, the median levels varied: 13,163.7 AU/mL and 7646.2 AU/mL in the COVID-19-infected group vs. 6788.0 AU/mL and 589.8 AU/mL in the non-infected group.

In Vietri’s study [19], blood was collected 30, 60, and 90 days after the second dose of the vaccine, and decreasing antibody levels were observed. The median IgG antibody (BAU/mL) levels were 1901.8, 1244.9, and 1032.4 after 30, 60, and 90 days following the second vaccination against COVID-19, respectively. All patients received the BNT162b2 vaccine.

Inchingolo et al. [20] collected blood three times. Two months after vaccination, 100% of the personnel had IgG titers ≥ 2000 BAU/mL. During the follow-up, the antibody levels declined. At the second collection titer (75 days after the first), only 1.7% of the patients showed anti-spike IgG levels under 500 BAU/mL. At the third titer (130 days after the second titer), 16.1% of the patients showed anti-spike IgG levels under 500 BAU/mL. All individuals received the BNT162b2 vaccine.

In Eyre’s study [21], the patients were encouraged to undergo serological tests before the first and second vaccinations and additionally approximately 4 weeks after the first vaccination. After being tested > 14 days after the first vaccination and prior to the second vaccination, 99.5% receiving the Pfizer–BioNTech vaccine were seropositive vs. 97.1% receiving the Oxford–AstraZeneca vaccine. In those receiving the Pfizer–BioNTech vaccine, the median anti-spike IgG reading > 21 days after the first dose of vaccine was 1028 AU/mL. In those receiving the AstraZeneca vaccine, the median was 435 AU/mL. After 21 days of the second vaccine dose (results only for Pfizer–BioNTech), the median IgG levels were 10,058 AU/mL (without evidence of infection) and 18,047 (10,884–22,413) AU/mL (with evidence) [21]. Further details regarding the studies included are presented in Table 2 and Table 3.

## 4. Discussion

Although more than 5 years have passed since the emergence and global transmission of SARS-CoV-2, our understanding of the evolution of protective immune responses to the virus is still limited [22].

Therefore, we do not fully understand why the COVID-19 pandemic ended, but it is likely due to a combination of factors. The end of the SARS-CoV-2 pandemic can be explained by a large portion of the global population acquiring immunity through natural infection or vaccination, i.e., so-called hybrid immunity. According to Ioannidis JPA [23], by the end of 2021, an estimated 73–81% of the global population was either vaccinated, infected, or a combination of both. In addition, the severity of COVID-19 relates to the virus variant [24]. On 24 November 2021, WHO announced the newly discovered Omicron variant (B.1.1.529). This variant, with new mutations in its spike protein, increases transmissibility and decreases antibody and vaccine responses, making it more virulent [25]. The subvariants that emerged after Omicron are highly infectious, and they no longer have the same level of virulence. All the studies in this systemic review ended before the Omicron variant appeared, so the increase in the incidence of COVID-19 was not noted in these studies.

Neutralizing antibodies (NAbs) serve as key effector molecules produced by B cells in response to viral infections or vaccinations and are considered a hallmark of the immune protection against SARS-CoV-2 infection [26].

Generally, broad neutralizing antibody assays show a strong correlation with IgG serological assays [26], which makes this review more realistic.

In this review of studies, approximately 11% of the participants had prior exposure to SARS-CoV-2. These results align with data from the Institute for Health Metrics and Evaluation (IHME), which indicate that 12% of individuals in the European Region had been infected with SARS-CoV-2 by early 2021 [27]. This suggests that our study population may be regarded as representative of the European context.

At the end of 2021, after a year of vaccine availability, almost 56% of the Polish population had been vaccinated. In 2021, there were 2,401,229 reported cases of COVID-19, compared to 1,294,878 cases in 2020. However, lockdown restrictions in 2021 were less strict than those in 2020 [2,28]. Additionally, in November 2021, WHO announced the newly discovered Omicron variant [25]. Today, it is difficult to predict how the situation might have unfolded without widespread vaccination efforts.

Many studies have examined antibody levels against SARS-CoV-2. However, it was challenging to find publications that did not focus on specific groups, such as HIV patients or organ transplant recipients. Ultimately, we selected only five studies conducted in the non-vulnerable population. Most excluded studies involved either highly specific study populations or vaccination regimens not approved by the FDA or EMA. Some studies lacked analyses of post-vaccination COVID-19 cases or did not measure antibody persistence over time. Additionally, certain studies excluded participants with prior COVID-19 infections before vaccination. Our aim was to analyze the general population, where many individuals had both prior infection and vaccination.

Due to differences in methodology, it was hard to compare the results between the studies. Each study had its own assessment points and different tests or types of antibodies which were analyzed. Generally, individuals with prior infection showed higher median IgG antibody levels compared to those without prior infection, which was confirmed in many studies [29,30]. This difference persisted over time, although a gradual decrease in antibody levels was observed in both groups.

We have several vaccines against SARS-CoV-2; unfortunately, in our review, we could compare only two different vaccines because all studies which focused on other vaccines failed to meet the inclusion criteria of our review. According to Eyre et al. [21], after the first dose of the Pfizer–BioNTech vaccine, the recipients produced higher antibody levels compared to the participants who received the Oxford–AstraZeneca vaccine, with a more than two-fold increase in individuals who had not been previously infected. All healthcare workers tested more than 14 days after their second vaccination were found to be seropositive, though this group only included 22 individuals who received the Oxford–AstraZeneca vaccine, so it is hard to draw a conclusion comparing these two vaccines over a long period of time.

No vaccine offers complete protection against symptomatic COVID-19. In the included studies, between 0.1 and 3.8% of the participants had COVID-19 after vaccination during the follow-up from days 144 to 302. Observational studies showed similar results. According to Naleway et al. [31], during the July–September 2021 surveillance period, SARS-CoV-2 infection occurred among 8.7 per 1000 fully vaccinated persons [31]. At the same time, in Europe, the incidence rate of the general population was around 7% [12]. Moreover, none of the studies presented here indicated a specific threshold that guaranteed a sufficient level of immunity to the infection. Instead, the likelihood of infection generally decreased as the immune response increased, but there was significant variation among individuals. This finding was consistent with research on respiratory syncytial virus, where a higher level of antibodies was linked to a lower risk of infection, yet cases still occurred even with elevated antibody levels. This suggests that an exact threshold for individual protection does not exist [32].

There is no doubt that vaccinations played a crucial role in the ending of the COVID-19 pandemic. Today, despite improved evidence-based medicine for COVID-19 and the availability of antiviral drugs, vaccination as a primary prevention strategy is the cornerstone of protection against SARS-CoV-2 infection, saving many lives [33].

## Figures and Tables

**Table 1:** The full search query.

Database	Search Terms	Date Searched Through	Number of Articles
PubMed	(((“COVID-19”[Mesh]) OR (“SARS-CoV-2”[Mesh])) AND ((“Immunogenicity, Vaccine”[Mesh]) OR (“Immunoglobulins, Intravenous”[Mesh]))) AND ((“Vaccination”[Mesh]) OR (“Vaccines”[Mesh])) NOT SYSTEMATIC REVIEW, NOT REVIEW	04 January 2023	676
Embase	50 AND ‘Article’/it AND (2020:py OR 2021:py OR 2022:py OR 2023:py) AND (‘clinical article’/de OR ‘controlled clinical trial’/de OR ‘controlled study’/de OR ‘diagnostic test accuracy study’/de OR ‘human’/de OR ‘major clinical study’/de)	04 January 2023	1041
Scopus	((TITLE-ABS-KEY (COVID-19)) OR (TITLE-ABS-KEY (Sars-CoV-2))) AND ((TITLE-ABS-KEY (immunogenicity)) OR (TITLE-ABS-KEY (“antibody levels”)))) AND ((TITLE-ABS-KEY (vaccination)) OR (TITLE-ABS-KEY (vaccine))) not “SYSTEMATIC REVIEW” AND (EXCLUDE (DOCTYPE, “re”))	04 January 2023	509

**Table 2:** Antibody levels against SARS-CoV-2 after vaccination.

Level of Antibodies Against SARS-CoV-2	[17]		[18]		[19]		[20]		[21]	
The first period of time: 42–51 days after first vaccination	IgA + IgG + IgM COV+ (n = 98) Me	2500 *	IgG n = 475 Me (IQR)	1314.1 (0.3–5712)	IgG n = 45 M (95% CI)	1901.8 (1698.4–2105.1)	Not estimated		COV + Median (IQR)	1436.3 (915.1–2225.1)
	IgA + IgG + IgM COV− (n = 1074) Me (IQR)	1659.0 (1611.5)	IgA n = 475 Me (IQR)	22.1 (0–64.6)					COV− Median (IQR)	2577.1 (1554.2–3200.6)
The second period of time: 81–90 days after first vaccination	Not estimated		IgG n = 377 Me (IQR)	306.8 (0–5712)	IgG n = 45 M (95% CI)	1244.9 (1067.3–1422.5)	IgG M (SD)	8413 (9510)		
			IgA n = 377 Me (IQR)	7.9 (0–43.4)						
The third period of time: 158–180 days after first vaccination	IgA + IgG + IgM COV+ (n = 91) Me	2500 *	IgG n = 322 Me (IQR)	96.46 (0–5712)	Not estimated		IgG M (SD)	3.880 (5.156)		
	IgA + IgG + IgM COV− (n = 1037) Me (IQR)	584.0 (607.0)	IgA	Not estimated						
The fourth period of time: 288–302 days after first vaccination	IgA + IgG + IgM COV+ (n = 69) Me (IQR)	2308.0 (1345.5)	Not estimated		Not estimated		IgG M (SD)	1.473 (1.818)		
	IgA + IgG + IgM COV− (n = 753) Me (IQR)	419.0 (526.5)								

* Results above the 2500 BAU/mL instrument limit were rounded to 2500 BAU/mL; all antibody titers are presented in BAU/mL. CI—confidence interval; M—mean; Me—median; SD—standard deviation.

**Table 3:** Basic characteristics of studies presenting vaccination and diagnostic tests included in this systematic review.

Ref	Date of First Blood Collection	Country	Study Sample	Age [Years]		Vaccine Product	Diagnostic Tests Which Were Used to Measure Antibody Levels
[17]	01.2021–02.2021	Italy	1172	766 females Mean (SD)	49.0 ± 16.7	BNT162b2 mRNA	ECLIA
				406 males Mean (SD)	52.0 ± 20.0		
[18]	12.2020–06.2021	Greece	511	Me (range)	54.0 (19–105)	BNT162b2 mRNA	CMIA, ELISA
[19]	01.2021	Italy	52	Range	25–70	BNT162b2 mRNA	CLIA
[20]	01.2021–10.2021	Italy	230	20–70		BNT162b2 mRNA	ELISA
[21]	12.2020–01.2021	United Kingdom	4315	BNT162b2 Me (IQR)	41 (30.51)	BNT162b2 mRNA or ChAdOx1-S	ELISA
				ChAdOx1-S Me (IQR)	42 (30.52)		

CLIA—chemiluminescence immunoassay, quantitative assay; CMIA—chemiluminescent microparticle immunoassay, quantitative assay; ELISA—enzyme-linked immunosorbent assay, quantitative assay; ECLIA—electrochemiluminescent assay, qualitative assay; Me—median.

## Data Availability

The original contributions presented in this study are included in the article/Supplementary Material. Further inquiries can be directed to the corresponding author.

## Supplementary Materials

The following supporting information can be downloaded at https://www.mdpi.com/article/10.3390/microorganisms13030535/s1: Table S1: Sensitivity and specify of the diagnostic tests.

## Abbreviations

The following abbreviations are used in this manuscript:

COVID-19	Coronavirus disease 2019
SARS-CoV-2	Severe acute respiratory syndrome coronavirus 2
