# Supplementary material for: SARS-CoV-2 Vaccine-Induced Humoral Immunity in Immunocompetent European Adults: A Systematic Review
Source: Microorganisms. 2025 Feb 27;13(3):535. doi: 10.3390/microorganisms13030535 (PMC11944475; doi:10.3390/microorganisms13030535)
Supplement: Supplementary file 1 [file microorganisms-13-00535-s001.zip › microorganisms-3451193-supplementary.pdf]

Table S1. Sensitivity and specify of the diagnostic tests

Ref	Diagnostic test	sensitivity	specifity
[15]	ECLIA “anti-SARS-CoV-2-S" test	98.8 % (95 % CI: 98.1 – 99.3 %)	100 % (95 % CI: 99.7 – 100.0 %)
[16]	ABBOTT SARS-CoV-2 IgG II assay	99.4% (95% = CI 96.5-99.9%)	99.6% (95%CI = 99.2-99.8%)
[17]	TrimericS IgG (DiaSorin TriS IgG; DiaSorin S.p.A) chemiluminescence immunoassay (CLIA)	sensitivity 0–7, 8–14 and ≥15 days post-RT-PCR: 46.7–82, 74.2–97.7 and 94.5–99.6%, respectively	99–99.7%
[18]	ELISA LIAISON® SARS-CoV-2 TrimericS IgG assay	The sensitivity of this assay unknown	The specifity of this assay unknown
[19]	Abbott SARS-CoV-2 IgG II Quant antibody test	99.4% (95% = CI 96.5-99.9%)	99.6% (95%CI = 99.2-99.8%)
